# Playing with Strangers: Which Shared Traits Attract Us Most to New People?

**DOI:** 10.1371/journal.pone.0129688

**Published:** 2015-06-08

**Authors:** Jacques Launay, Robin I. M. Dunbar

**Affiliations:** Department of Experimental Psychology, University of Oxford, Oxford, United Kingdom; University of Tuebingen Medical School, GERMANY

## Abstract

Homophily, the tendency for individuals to associate with those who are most similar to them, has been well documented. However, the influence of different kinds of similarity (e.g. relating to age, music taste, ethical views) in initial preferences for a stranger have not been compared. In the current study, we test for a relationship between sharing a variety of traits (i.e. having different kinds of similarity) with a stranger and the perceived likeability of that stranger. In two online experiments, participants were introduced to a series of virtual partners with whom they shared traits, and subsequently carried out activities designed to measure positivity directed towards those partners. Greater numbers of shared traits led to linearly increasing ratings of partner likeability and ratings on the Inclusion of Other in Self scale. We identified several consistent predictors of these two measures: shared taste in music, religion and ethical views. These kinds of trait are likely to be judged as correlates of personality or social group, and may therefore be used as proxies of more in-depth information about a person who might be socially more relevant.

## Introduction

There is a well-established tendency for friends to be more similar to one another than would occur by chance, and this is known as homophily(for reviews see [1,2]). Several studies have demonstrated a robust directional relationship between believing that an interaction partner has similar attitudes to oneself, and attraction towards that partner [3–12]. People express a greater desire to engage with someone they think is more similar to them [3,13–16], have closer existing relationships with people who are more similar to them [17–19], are more likely to maintain relationships with those with whom they perceive similarities [20–22] and are more likely to behave altruistically towards friends with whom they have more in common [19]. Choice homophily, which is the tendency to choose to associate with people who are more similar to oneself, is particularly interesting because it can indicate a directional relationship with attraction, and cognitive biases towards certain individuals or groups, unlike forms of homophily that occur between people by chance, or homophily identified in existing relationships. Here we are specifically interested in the directional relationship from identifying similarities with another person to attraction towards further interaction with that person.

Typically, experimental manipulation of similarity when investigating whom people are interested in interacting with has involved questionnaires about attitudes, for example asking participants whether they agree that having money is an important life goal. By asking participants to rate statements about an interaction partner, and varying the proportion of traits that they share with that partner, it has been shown that relative number of shared attitudes have a consistent positive relationship with interpersonal attraction [3] of a kind likely to cause people to pursue friendships with one another outside experimental settings. A less well understood aspect of attraction towards strangers is whether different shared traits have different effects, and here we ask whether choice homophily is specific to some traits, or whether it could occur as a more general ‘in-group’ identification effect.

In-group membership has generally been studied independently from choice homophily. There is a tendency to favor in-group members over out-group members, even in ‘minimal group’ situations, i.e. between groups that have been assigned arbitrarily and therefore have the least possible sense of social identity [23–30]. However, the relationship between this effect and choice homophily as a consequence of shared traits remains unclear, especially as they focus on different trait types. In the case of arbitrary group assignment, transforming the dichotomous group distinction (i.e. group A vs. group B) into a three-group distinction (i.e. group A vs. group B vs. group C) *reduces* the tendency towards in-group favoritism [31,32]. Real-world categories that act as markers for groups of individuals, such as age, are rarely dichotomous, so in these cases we might expect minimal group effects to become so diluted that they become irrelevant [24].

While dilution of in-group effects with increasing complexity of trait category could occur, evidence shows that in fact real social categories *do* define the kinds of social networks that we interact in [33]. Studies in relational demographics (a research field that looks more specifically at similarities between people who form friendships in the workplace) have typically demonstrated that people are likely to share demographic characteristics with people that they interact with, including age for example [34,35]. Curry & Dunbar [19] recently showed that within social networks, there is a linear relationship between social proximity of a friend and a variety of traits that are shared with them. However, as this study looked at existing friendships, it could not demonstrate a causal relationship in which different shared traits preceded the establishment of a relationship with a stranger. As there is a tendency to lose contact with friends with whom we have less in common [20,22], and to conform with the norms of our social groups [36,37], it is still unclear which traits have a causal influence on willingness to pursue a friendship with a given individual, especially when time or distance place a maintenance stress on those relationships [38]. A stochastic agent-based modeling framework has also been used recently to infer which features might determine friendship formation using online social network data [39], but this did not directly manipulate shared traits to determine which could be most influential when interacting with a stranger.

Different forms of similarity have been measured when studying choice homophily and minimal group membership. Experiments on choice homophily have relied on statements about attitude (e.g. ‘money as one of the most important goals’[4] ‘death penalty for murder’ [40]). Given that minimal group membership research demonstrates that changes in social closeness occur due to arbitrary categorization, the kinds of similarity are thought to be relatively unimportant. In contrast, research on relational demography has used basic demographic information (such as age, gender, race [35]), and homophily has typically looked at the effect of shared values. Only a few studies have combined different types of trait in similarity-attraction paradigms, and these have typically been typically interested in just one trait (notably race [41,42]).

Here we combine different trait types in order to determine whether there is a consistent relationship between some particular traits and positive evaluation of a potential social partner. Traits were selected that indicate status homophily (based on formal, informal or ascribed status) and value homophily (based on traits such as shared hobbies, interests or tastes [33,43]). Status homophily can be further separated into two main types: (1) major socio-demographic factors that we are born with and remain unchanged throughout life (e.g. age, race) which also tend to be perceptually salient and (2) characteristics that we acquire later in life and are more variable, such as religion, education, or occupation, which are more cognitive and relate more closely to value traits. While studies in relational demography have typically focused on socio-demographic features and homophily studies more generally focus on values, there is as yet no experimental evidence assessing whether and how these different forms of similarity interact to influence attraction towards strangers during the initiation of friendships.

Another important distinction to make here is between choice homophily and induced homophily [44]. While choice homophily is a consequence of actively choosing to interact with a person with whom we share characteristics, induced homophily is that which occurs as a consequence of the people we meet and have the opportunity to interact with. In particular, this distinction is important when connecting the field of relational demographics with homophily research. As relational demography looks at people who work together, it primarily identifies induced homophily and has tended to show more similarities in status traits (age, gender, class), while homophily research has focused on value traits. In this study, we use an online design, in which participants have no prior knowledge about a partner, to ensure that we are solely measuring choice homophily, meaning that our results relate to whom people are initially most attracted to rather than friendships that occur through forced convenience.

There is currently some contention over the measurement of attraction [45], so here we will evaluate several potential measures that are relevant to an online experimental paradigm. While we primarily focus on rating scales (e.g. “how much do you think you would like your partner if you met them?”), we will also assess whether participants conform with the behavior of a partner, using an estimation task [46]. As a third measure of affiliation, we use the Inclusion of Other in Self Scale [47]. Since choice homophily has been shown to be a robust effect, we first assess which of these measures demonstrate the expected effect, then test which traits contribute most towards the values of those measures. In Experiment 1, we test five measures in order to evaluate which demonstrate the well-documented positive linear relationship with increased proportion of shared traits. We then use these same measures in Experiment 2 to determine which traits are most important in attraction to similar others.

In line with an extensive existing literature, we expected participants to feel more positive towards partners that they believed they shared more traits with. Beyond this primary hypothesis, we tested which traits are the best predictors of positive evaluation of a partner. If we find no consistent relationship between trait types and positive evaluation of a partner, then similarity-attraction may act purely as a minimal group membership effect.

## General Method

### Ethics Statement

All participants gave written informed consent and the experiment was approved by the University of Oxford Central University Research Ethics Committee (Ref. MSD-IDREC-C1-2013-061).

### Participants

Participants were recruited via Maximiles, a commercial online survey management system, and completed the study online using their own computers. All participants received 100 Maximiles points (worth approximately £1.50) for their 10 minute participation.

### Design

A within-subject design was used to test whether sharing behavioral traits with others could influence attraction. Participants were introduced to a series of partners (whom they were told were human but who in fact were computers) with whom they shared varying numbers of traits, and were asked a number of questions to determine how positively they felt about each partner.

The independent variables were partner type (i.e. partners with whom they shared many traits vs. partners with whom they shared few traits), and which traits were shared. Dependent variables were a conformity task (outlined in the Procedure below), and a set of questions designed to assess positivity felt towards the partner, which might lead to attraction in real settings of friendship initiation.

### Procedure

Participants read an information sheet, provided consent, and were asked to read instructions which included a practice of the conformity (estimation) task. They then completed a survey with limited answer options provided using drop-box menus. After completing the questionnaire, participants were provided with an answer profile that they were told corresponded to a partner. The order of different partner types (sharing different numbers of traits) was counterbalanced between participants. Participants were told to read through this profile, and informed that they would be asked to recall some of these traits later in the experiment. The traits that would be shared with each partner were randomly determined. For traits that were not shared the computer partner was randomly assigned one of the other possible answers from the drop-box menu. Any traits that were shared with one partner would not be shared with another partner, which meant that all participants experienced sharing each of the possible traits.

In order to measure conformity we used a task previously developed by Castelli et al. [46]. Participants were shown a screen with a total of 60 X’s and O’s, with the number of each randomly determined. They were given five seconds to look at this screen, and were then asked to estimate how many O’s had appeared on the screen. Participants were given information about their partner’s guess before they were required to make their own guess, which was actually always the correct number of O’s. This was presented to participants as the main task of the experiment and they did not engage in any other form of interaction with partners.

Following this estimation task, participants were given a set of further questions, and asked to rate them on a standard Likert scale (1–7). The relevant questions were:

- How willing would you be to work with the same partner again on a different task? (1 = very unwilling, 7 = very willing).
- How much do you think you would like your partner? (1 = dislike a lot, 7 = like a lot).
- If you discovered your partner had cheated during the estimation task, how likely would you be to report it to the experimenter? (1 = very unlikely, 7 = very likely).

After answering these questions participants completed an Inclusion of Other in Self Scale (IOS [47]). This provides seven different images of circles that overlap one another to a greater or lesser extent, and asks which picture best represents how close one feels to a partner (“Please indicate below which of these pictures indicates how close you feel to the partner you interacted with:”). Participants were finally asked to recall four of the traits of their partner. This task has been widely used and provides a reliable index of how engaged an individual feels with someone else.

The same tasks were repeated for each of the partners. Following this procedure participants were asked to complete a very brief personality-type questionnaire [48], and to give their current home town. Finally participants were asked to read a full debrief of the experiment, and were given the opportunity to withdraw their data from the study without any penalty.

## Experiment 1

### Participants

In total, 294 participants were tested from a UK population (*N* female: 168, *Mdn* age group: 36–45), and all details as entered into the survey are given in S1 Table. All data are provided as DataExp1 at doi 10.5287/bodleian6ycl.4.

### Design

The experiment used a within-subject design, with three different partner types as the independent variable. Partners either shared nine out of fourteen traits (Partner A), five out of fourteen traits (Partner B), or no traits (Partner C) with the participant.

### Procedure

The procedure was as given above, and all questions asked are given in S1 Table, along with proportions of participants choosing each option. Questions assessed both demographic and value based traits: age, gender, ethnicity, natal location, religion, current location, occupation, income, highest level of education, music taste, political views, hobbies, sense of humor and ethical beliefs. The final two traits were assessed by giving participants a list of jokes/ethical statements that have been shown to divide opinions (jokes [49], ethical statements [50]) and asking participants to choose their favorite joke, or the ethical statement they agreed with most.

### Results

Questionnaire and estimation task data were first assessed to determine which dependent variables were most relevant to sharing traits with another person. Estimates in the conformity task were subtracted from the answers given by the computer partner, with absolute values of these used as an indicator of estimate proximity. These values were log-transformed to normality for use in statistical tests. IOS picture ratings were transformed to numerical values from 1 to 7, with 1 representing most separate. These values were negatively skewed, and were binned into ratings of 1, 2–3, 4–5 and 6–7 for statistical analysis, but non-parametric tests on raw data gave comparable results to those reported here. We first tested which of the five measures of attraction would best demonstrate a linear relationship with shared traits, as should occur in an indicator of choice homophily. We used a repeated measures MANOVA to test the effect of partner type (A, B or C) on the estimation task results, ratings of desire to interact again, likeability and reporting of cheating of a partner, and the binned IOS scale ratings. This showed significant differences between partner type and the combined measures, Pillai’s Trace = 0.15, *F*(2, 1166) = 9.5, *p* < .001. Univariate comparisons demonstrated significant differences by partner type for ratings of interaction with a partner again, *F*(2, 586) = 10, *p* < .001, *Gη* ^*2*^ = 0.03, partner likeability, *F*(2, 586) = 33, *p* < .001, *Gη* ^*2*^ = .10 (Greenhouse-Geisser corrected), and binned IOS scale ratings, *F*(2, 586) = 33, *p* < .001, *Gη* ^*2*^ = .10 (Greenhouse-Geisser corrected). No significant differences between partner types were found in the estimation task results (*p* = .95), or ratings of reporting cheating to the experimenter (*p* = .22).

Follow-up paired *t*-tests on future interaction with a partner demonstrated significantly more favorable ratings of Partner A (*M* = 5.0, *SD* = 1.5) than Partner B (*M* = 4.7, *SD* = 1.5; *t*(293) = 3.8, *p* < .001, Cohen’s *d* = 0.22), and between Partner A and Partner C (*M* = 4.7, *SD* = 1.4; *t*(293) = 4.1, *p* < .001, Cohen’s *d* = 0.24). No significant differences were found between Partner B and Partner C, *t*(293) = 0.18, *p* = .85).

**Fig 1:**
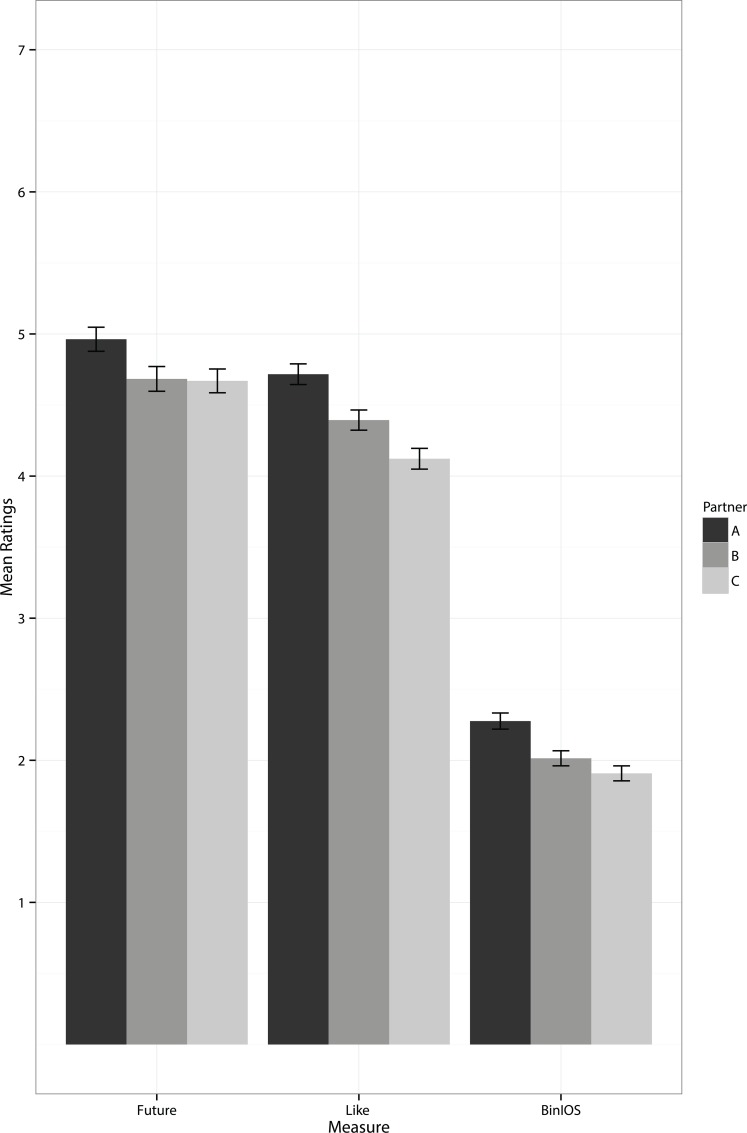
Mean ratings for Partner A (sharing nine of fourteen traits), Partner B (sharing five of fourteen traits) and Partner C (sharing no traits) on desire for future interaction, likeability and binned IOS scale. Error bars show standard error.

Paired *t*-tests on partner likeability demonstrated the predicted significantly higher ratings of Partner A (*M* = 4.7, *SD* = 1.2) compared with Partner B (*M* = 4.4, *SD* = 1.2; *t*(293) = 4.283, *p* < .001, Cohen’s *d* = 0.25), between Partner B and Partner C (*M* = 4.1, *SD* = 1.3; *t*(293) = 4.1, *p* < .001, Cohen’s *d* = 0.24), and between Partner A and Partner C, *t*(293) = 7.7, *p* < .001, Cohen’s *d* = 0.45. Similarly, paired *t*-tests on the binned IOS scale ratings showed significantly closer ratings of Partner A (*M* = 2.3, *SD* = 0. 98) compared with Partner B (*M* = 2.0, *SD* = 0.91; *t*(293) = 5.2, *p* < .001, Cohen’s *d* = 0.31), of Partner B compared to Partner C (*M* = 1.9, *SD* = 0.90; *t*(293) = 2.5, *p* = .013, Cohen’s *d* = 0.13), and Partner A compared to Partner C, *t*(293) = 7.6, *p* < .001, Cohen’s *d* = 0.49. These results are summarised in Fig 1, and demonstrate that only the measures of likeability and IOS scale showed the expected relationship, with increased attraction towards people with whom more traits were shared. This means that only these measures appear to index the choice homophily relationship that we are expecting, so these measures will be used to determine which traits contribute most towards positive ratings of strangers.

Following this, we tested which traits had the largest influence on positive evaluation of a partner. Partner likeability, and IOS scale ratings were each used to measure the importance of sharing different types of trait with a partner because they seem to best represent choice homophily. Each trait was coded for whether it was shared with a partner or not using a 1 or 0 respectively. These coded variables were then entered into a multilevel linear model, using individual IDs as Level 2 predictive factors with random intercepts (effectively normalising for individual variability in baseline response and removing the problem of non-independence of data points), and traits as Level 1 predictive factors with fixed intercepts and fixed slopes. All instances in which participants recalled less than one out of four features of their partner correctly were excluded from this analysis on the basis that in these trials participants had not remembered a sufficient amount of information from the profile given (43 data points out of a total of 882). The statistics program R version 3.0.1 was used to perform modelling, with the package lme4 version 0.999999–2. All 14 factors were entered into a model for each dependent variable, with each factor acting as an additional predictor with no interaction terms, so the model for likeability would be given by (each letter represents a coefficient):

$$\begin{array}{l} \text{Likeability}=\text{General Constant}+\text{Individual constant}\;\left(\text{for each participant}\right)+\text{Age}\\ \;\times \text{a}+\text{Sex}\times \text{b}+\text{Ethnicity}\times \text{c}+\text{Natal Area}\times \text{d}+\text{Religion}\times \text{e}\\ \;+\text{Area}\times \text{f}+\text{Occupation}\times \text{g}+\text{Income}\times \text{h}+\text{Education}\times \text{i}\\ \;+\text{Musical taste}\times \text{j}+\text{Political views}\times \text{k}+\text{Hobbies}\times \text{l}\\ \;+\text{Preferred Joke}\times \text{m}+\text{Ethical Statement}\times \text{n} \end{array}$$

**Fig 2:**
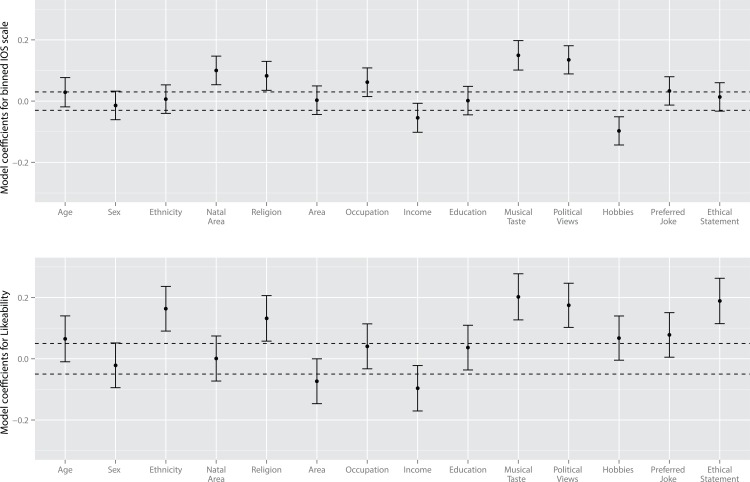
Results of multilevel linear modelling from Experiment 1. Dashed lines indicate approximate boundaries for significance, predictors with standard errors that do not cross this line have t > 1.6.

The resulting coefficients are given in Fig 2 and Table 1, along with standard errors to indicate which act as significant predictors of each dependent variable (*t* > 1.6 is used as the criteria for reporting significance).

**Table 1:** Multilevel model coefficients in Experiment 1 and Experiment 2.

Experiment	Predictor	Binned IOS Coefficient (SE)	Likeability Coefficient (SE)
1	Constant	1.88 (0.05)*	4.10 (0.07)*
	Age	0.03 (0.05)	0.07 (0.07)
	Sex	-0.01 (0.05)	-0.02 (0.07)
	Ethnicity	0.01 (0.05)	0.16 (0.07)
	Natal Area	0.10 (0.05)*	0.00 (0.07)
	Religion	0.08 (0.05)*	0.13 (0.07)*
	Area	0.00 (0.05)	-0.07 (0.07)
	Occupation	0.06 (0.05)	0.04 (0.07)
	Income	-0.05 (0.05)	-0.01 (0.07)
	Education	0.00 (0.05)	0.04 (0.07)
	Musical Taste	0.15 (0.05)*	0.20 (0.08)*
	Political Views	0.13 (0.05)*	0.17 (0.07)*
	Hobbies	-0.10 (0.05)*	0.06 (0.07)
	Preferred Joke	0.03 (0.05)	0.08 (0.07)
	Ethical Statement	0.14 (0.05)	0.19 (0.07)*
2	Constant	1.90 (0.10)	4.15 (0.13)
	Ethnicity	0.02 (0.06)	0.10 (0.09)
	Natal Area	0.01 (0.06)	0.08 (0.09)
	Religion	0.12 (0.06)*	0.22 (0.09)*
	Area	0.04 (0.06)	0.11 (0.09)
	Musical Taste	0.11 (0.06)*	0.17 (0.09)*
	Political Views	0.10 (0.06)	0.13 (0.09)
	Ethical Statement	0.16 (0.06)*	0.22 (0.09)*

* indicates significance at *t* > 1.6.

The most important predictor of likeability and IOS scale ratings was taste in music, with political views second most important. Religion also acted as a significant predictor of both ratings, while choice of ethical statements, ethnicity, and natal area were only predictors of one of the measures. Given that the results are directly modelling ratings using coding for whether a trait was shared or not they can be interpreted directly in terms of the contribution each trait made towards likeability (i.e. effect size). So coefficients of up to 0.2 suggest that when a trait was shared it caused a 0.2 larger likeability rating compared with when it was not shared on the likeability scale (which ranged from 1–7). The individual coefficients (for the five variables that differ significantly from zero (ethnicity, religion, musical taste, political views and ethical statement) range from 0.13–0.18; adding these five significant predictors of likeability together suggests that, on average, individuals who share all these traits will rate their partner 0.84 higher than those who do not. Although this is a small effect, it is of a magnitude that we might expect given the contrived setting in which the experiment was conducted; critically, the valence of each of the different traits is of more importance than the effect size per se in this paradigm. An alternative way to express the effect here is against baseline ratings (i.e. the model constant), which are indicative of how participants rate someone with whom they share no traits. Against this baseline the five significant predictors of likeability in Experiment 1 increase ratings overall by 20% (i.e. 0.84/4.1), or sharing taste in music alone could be said to increase likeability of a stranger by 5% (0.2/4.1). Significant positive predictors of the binned IOS measure similarly increase baseline ratings by 25%, or shared musical taste can increase baseline ratings by 8%.

Note that shared hobbies acted as a negative predictor of IOS ratings, suggesting that having hobbies in common with a stranger made people view them as less closely linked to themselves; however, this result was not replicated in likeability ratings and should be interpreted with caution. Other than this negative effect of hobbies, the overall pattern of results suggests that value traits were important predictors of positivity expressed towards a stranger.

## Experiment 2

In Experiment 1, we tested which measures of attraction would demonstrate the predicted linear relationship with shared traits, then tested which traits were most relevant predictors of these measures. Experiment 2 was designed to focus specifically on the traits that were shown to be the most important predictors of homophily in Experiment 1. By doing this, we both ensure that the modelling results are consistent, and check that, in a simpler situation with fewer shared categories and fewer partners, participants are influenced by similar factors. We here report only results directly relating to this question, using shared traits to predict ratings of likeability and ratings of IOS scale, as these measures were shown to best reflect the expected linear relationship with shared traits. In Experiment 1,three partners were required in order to determine which measures would demonstrate the expected linear relationship with number of shared traits. The design of Experiment 2 meant that only two partners were necessary, making a simpler design possible.

### Participants

195 participants were tested (108 Female, Age, M = 45.0, SD = 13.6). All data are provided as DataExp2 at 10.5287/bodleian6ycl.4.

### Design

The study used a within-subject design. Participants interacted with two partners: with one they shared five out of seven traits, and with the other they shared two out of seven traits. Traits were selected from the most important positive predictors from each category in Experiment 1: ethnicity, natal area, religion, current area, musical taste, political views and ethical statement.

### Procedure

The procedure was as outlined in the general methods. Participants entered information about seven traits during the survey part of the experiment. They then interacted with two partners. As less traits were included in the initial survey, participants were only required to remember three of their partners’ traits in the test. Numbers of possible answers for traits given in drop-down menus differed between participants, but that difference is not relevant to the current question and further details relating to this are reported as Experiment 1 in Launay & Dunbar [51].

### Results

IOS scale ratings were binned as in Experiment 1. Results for these binned measures and ratings of partner likeability were then modelled using multilevel linear models, with each of the seven shared trait as a potential predictor of ratings, coded using a 1 or 0 for whether the trait was shared or not. Participant IDs were included as Level 2 predictors with random intercepts and traits were modelled as Level 1 predictors with fixed intercepts and fixed slopes. If participants recalled less than one out of four features of their partner correctly these data were excluded from this analysis (30 data points out of a total of 390).

**Fig 3:**
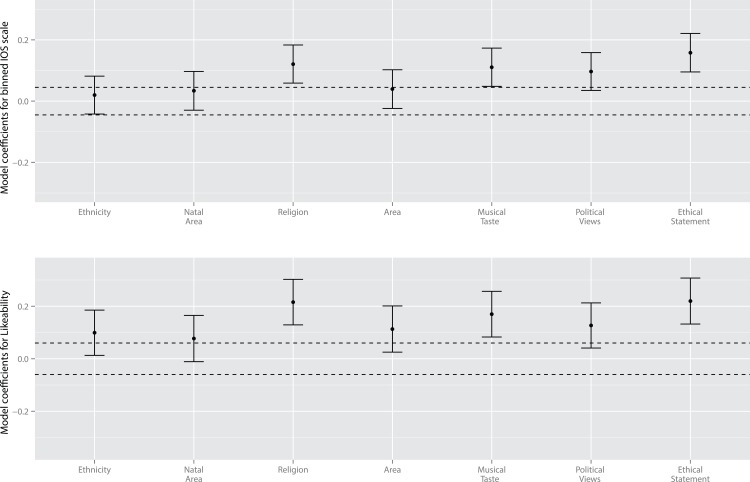
Results of multilevel linear modelling from Experiment 2. Dashed lines indicate approximate boundaries for significance, predictors with standard errors that do not cross this line have t > 1.6.

Results from the models are given in Fig 3 and Table 1. These show a similar pattern to those of Experiment 1, with ethical statements, religion and musical taste acting as significant predictors of the likeability and IOS ratings of the partner. Unlike Experiment 1, shared political views were not a significant predictor of likeability or IOS ratings of the partner, and neither were the current region, natal region or ethnicity. Calculating the cumulative effect of significant positive predictors against baseline ratings gives slightly lower values than in Experiment 1. In Experiment 2 sharing the three significant predictors of likeability with a partner (ethical statement, religion and musical taste) increased participant ratings 15% over baseline, while sharing the ethical statement (the most important predictor of likeability in this experiment) increased ratings by 5% over baseline. For the binned IOS ratings sharing the three significant traits increased partner ratings by 21%, and sharing ethical statements increased partner ratings by 8%.

Value traits generally acted as better predictors of likeability and IOS scale ratings than status traits. Results from both Experiment 1 and Experiment 2 suggest that sharing taste in music and religion play a consistent role in determining the likeability of a similar stranger, while other value traits (political views and ethical statements) also tend to be predictive of measures of affiliation.

## Discussion

This experiment explores the types of homophily that have most potential to influence formation of social bonds. Results from Experiment 1 first demonstrate that ratings of partner likeability and the IOS scale have a positive relationship with increased proportion of shared traits, at least in the context of online introductions. Using these measures as indices of choice homophily, it was possible to show that value traits such as music taste, political interests and ethical values were the most important predictors of initial positivity towards a stranger. Having the belief that we would like someone more, or feel greater connections with them, is likely to be a key stage in the initiation of a friendship. We do not, of course, suggest that these are the only traits that people use when choosing friends: we have not, for example, considered personality traits, for which there may well be choice homophily. Our purpose has not been to examine in detail all the possible criteria that individuals might use for these purposes; rather, our purpose has been to ask whether a particular set of criteria that have been shown to underpin existing friendships as well as willingness to behave altruistically towards an existing friend also generalise to the willingness to behave positively towards a complete stranger.

One of the classic findings from social psychology has been that we can be made to feel a sense of belonging to almost any arbitrarily pre-constituted group [24]. Here we demonstrate that when we can, in effect, choose whether or not to belong to a group (by forming a friendship with someone), we use some traits more than others to determine whom we might prefer to associate with [33], as indexed by ratings of how much we might like a partner or include them in our sense of self. This suggests that choice homophily effects are more complex than minimal group membership effects, and implies that even when engaging in quite simple activities such as the online task used here participants were using all the information available to try to determine the personality and character of prospective partners. Two points are worth making. First, if the current results solely reflected a form of minimal group membership, we would not expect some traits to consistently act as better predictors of likeability and IOS because arbitrary shared group membership is known to be sufficient to cause this effect. The fact that the various traits influence the outcome differently implies that something else other than minimal group membership is at stake here. Second, we cannot use the dependent measures to determine whether effects are related to group membership or interpersonal attraction: both effects would be expected to result in a more positive attitude towards a target individual.

It is possible that the online paradigm had some influence on which traits were most important. Given that participants did not have visual access to a partner, they might as a result be less strongly affected by features such as age, which can be more apparent during physical interaction. However, the current aim was to compare all of the potential trait types while holding all other features constant (i.e. providing no privileged access to one form of information over any other). Physical trait characteristics certainly can play an important role during real interactions, but they are by no means the only traits that we use as the basis for friendship formation (note that we specifically exclude romantic relationships). We here build on extensive research showing that friendships emerge out of suites of shared cultural interests, and our question focusses on whether some of these are more important than others, independently of whatever other physical factors might play a role.

Although sharing a feature such as an occupation may be just as likely to give people a starting point in a conversation with a stranger, our results suggest that people do not value this form of homophily as much as information about tastes. The influence of homophily on initiation of relationships is not simply about experiencing some broad categorization of membership, or about giving us some starting point for a relationship, but specifically about the belief that we share valuable character traits with another person. Given the kinds of traits that were the most consistent predictors in the current study, it is possible that either identification of specific personality characteristics is involved, or that tribal traits (i.e. those which indicate membership of a particular cultural group [52,53]) are the most likely to encourage attraction to a stranger. An important point in the current study is that by investigating the initiation of relationships with strangers online we are specifically targeting choice homophily, rather than induced homophily. While many demographic features such as gender and age normally exhibit homophily, this does not have to be the case when people are able to choose their friends.

Sharing music taste with strangers was found to be a particularly good predictor of whether participants would feel close to those strangers, and expect to like them more. It has been argued that there are close relationships between music taste and other features of personality and social identity [54–56]. It is therefore likely that people assume that identity is prominently signaled by this form of group membership, and that music taste can give us a lot of information about the relationship that we might be able to develop with a stranger. One perspective on the function of music in our evolutionary history is that it may have developed to encourage the formation and consolidation of social bonds [57,58], and the current result concords with this suggestion in that it suggests that those who share tastes in music are likely to have a stronger sense of solidarity.

Similarly, political views and statements about ethical beliefs are likely to give some insight into the character of another person and signify group membership, so the relevance of these traits as predictors of affiliative behavior also indicate that it is these underlying values that drive choice homophily. While any form of religiosity has been shown to have similar relationships with values (e.g. relating to hedonism, benevolence, achievement [59]), there are many differences in the extent to which these values are taken on by people of different religions [60]. Overall, it seems likely that all of the most important predictors in the current experiment (musical taste, political views, ethical views and religion) give some insight into a person’s viewpoint and social group. In contrast, traits such as general hobbies do not so clearly relate to values, and may thus reflect personal interests as opposed to communal allegiances, leading to a less positive relationship with partner ratings. It is possible that using categories of hobbies that are more clearly related to social groups (e.g. particular sports that are associated with different classes) would have caused this trait type to assume more importance in predicting measures of positivity, but here we did not use categories with any strong associations of this type.

From an evolutionary perspective it has been argued that homophilic assortment might indicate an attempt to associate with (and support) those with whom we are more closely related [40]. It has previously been demonstrated that even quite subtle cues of kinship (e.g. facial resemblance, similar names) can lead to more positive behavior towards a stranger [61,62]. The current experiments were not designed to specifically test the relative influence of traits that might better indicate kinship or other forms of group membership, and it is indeed likely that group membership in our evolutionary history was closely tied to our kinship networks. It is, however, relevant that traits such as musical taste and religion, within broad parameters, have relatively arbitrary and limitless sets of rules, which makes them particularly good signals of group membership [63].

The most significant predictors of positive partner ratings tend to match those previously identified when looking at emotional closeness and shared traits in established relationships [19]. In this previous experiment, sharing a sense of humor, sharing moral beliefs, sharing hobbies or interests, liking similar music, having similar personalities, and coming from the same area were the best predictors of emotional closeness with social network members. An important difference with the current experiment is that we investigate measures that could lead to the initiation of *new* relationships, and this allows us to make some causal inferences about how relationships might be formed. Two predictors of established relationships that did not seem to be relevant for the initiation of relationships with strangers were a shared sense of humor and shared hobbies/interests. This may indicate that homophily based on these traits becomes more important in the maintenance phase of relationships rather than at the initial stages of friendship formation, although this suggestion requires further investigation.

The independent variables used in the present study did not all yield the same results. Ratings of wanting to interact with a partner again showed some change according to shared values, but not as would be expected in typical cases of choice homophily. The number estimation task, generally used to measure conformity, and the question about reporting cheating to the experimenter did not demonstrate any differences between the three partners with different degrees of homophily. It is possible that the number task was subject to a ceiling effect as a consequence of participants making the same choice as the virtual partner. If so, this would make the measure inappropriate for the current purpose. This aside, given that there is a well-established relationship between sharing traits and the desire to affiliate [3], our finding that neither of these traits correlated with desire to affiliate most likely indicates that these measures are not germane to the establishment of new social relationships, but may be more relevant when measuring the strength of established relationships. We used the IOS scale and likeability ratings in our models in order to test the importance of different trait types because these two measures have been shown to have linear relationships with greater proportion of shared traits, as is well-documented in the literature on this effect (for a meta-analysis see [2]). An online experimental paradigm does not, of course, mimic in its full richness our experience of real life interactions, although it probably does mimic rather closely most people’s online social experience. However, our two key indices do measure the strength of real world social relationships extremely well, and we use the similarity of results with other findings in homophily research as evidence that these two measures can index interest in engaging with a partner (i.e. choice homophily), and hence can be used to assess the importance of different traits on choice homophily.

Although individual effect sizes were modest, we ought perhaps to expect this given that forming relationships with strangers is necessarily a complex process based on evaluating the many dimensions of another individual. In any case, raters invariably tend towards the median in Likert-type rating scales, and the magnitude of the difference between two sets of ratings should not be confused with the actual magnitude of an effect. The important question is simply whether or not there are statistically significant differences between the ratings of different partners, despite the fact that rating scales are inevitably imperfect. Our results consistently show that there are such differences, in two different experimental contexts.

In conventional experimental designs, we might seek to examine the impact of a single variable of interest while holding all others constant. Here, we were concerned with the predictive value of a number of different trait dimensions simultaneously, each of which is known to affect friendship quality. Assessing the importance of such a variety of different traits on initial attraction towards another person would be hard to achieve in lab-based experiments, in which only a small (and often geographically and socioeconomically discrete) population is sampled. Although the online paradigm used for the current experiment is far removed from interaction as it would occur in real life, it does provide some insight into initial preferences in a controlled manner, allowing careful manipulation of the trait information which would be much harder in other experimental or real life contexts. That said, of course, in the contemporary world, meeting new friends, and even romantic partners, online is becoming increasingly common. In such online contexts, one has little more to go on that the kinds of information provided in Experiment 1.

In conclusion, the current experiments demonstrate that the present subsample of a UK population were most influenced by sharing musical taste and religious beliefs with another person in determining how likeable a stranger might be, and how much they included them in their sense of self. Importantly, as different kinds of similarity do not have the same influence on these measures, minimal group membership does not sufficiently explain choice homophily. Nonetheless, some traits turn out to have particular salience over others. In particular and perhaps surprisingly, in the current sample people appear to take musical taste as an important indicator of personal features, and use this as a relevant predictor of whom they might want to engage with.

## Supporting Information

S1 Table Questions and answer options for the fourteen studied traits in Experiment 1, including numbers and proportions of people who chose each answer option. (DOCX)
