# Supplementary material for: Playing with Strangers: Which Shared Traits Attract Us Most to New People?
Source: PLoS One. 2015 Jun 8;10(6):e0129688. doi: 10.1371/journal.pone.0129688 (PMC4460138; doi:10.1371/journal.pone.0129688)
Supplement: S1 Table — (DOCX) [file pone.0129688.s001.docx]

**S1 Table**

S1 Table. Questions and answer options for the fourteen studied traits in Experiment 1, including numbers and proportions of people who chose each answer option.

| Question | Answer | Number of responses | Percentage of responses |
| --- | --- | --- | --- |
| Age: |  |  |  |
|  | 18-25 | 21 | 7.1 |
|  | 26-35 | 63 | 21 |
|  | 36-45 | 66 | 22 |
|  | 46-55 | 71 | 24 |
|  | 55+ | 73 | 25 |
| Sex: |  |  |  |
|  | Female | 168 | 57 |
|  | Male | 126 | 43 |
| Ethnicity: |  |  |  |
|  | Asian | 10 | 3.4 |
|  | Black | 7 | 24 |
|  | Indian | 5 | 1.7 |
|  | Hispanic | 2 | 0.68 |
|  | Middle-Eastern | 0 | 0.0 |
|  | White | 262 | 89 |
|  | Other | 8 | 2.7 |
| Area you grew up in: | |  |  |
|  | North England | 77 | 26 |
|  | Northern Ireland | 2 | 0.68 |
|  | Midlands | 49 | 17 |
|  | Scotland | 24 | 8.2 |
|  | Southeast England | 74 | 25 |
|  | Southwest England | 29 | 9.9 |
|  | Wales | 10 | 3.4 |
|  | Outside the UK | 29 | 9.9 |
| Religion: |  |  |  |
|  | Agnostic | 36 | 12 |
|  | Atheist | 58 | 20 |
|  | Buddhist | 5 | 1.7 |
|  | Christian | 146 | 50 |
|  | Hindu | 2 | 0.68 |
|  | Jewish | 3 | 1.0 |
|  | Muslim | 8 | 2.7 |
|  | Sikh | 1 | 0.34 |
|  | Other | 35 | 12 |
| Current location: | |  |  |
|  | North England | 74 | 25 |
|  | Northern Ireland | 3 | 1.0 |
|  | Midlands | 55 | 19 |
|  | Scotland | 24 | 8.2 |
|  | Southeast England | 90 | 31 |
|  | Southwest England | 28 | 9.5 |
|  | Wales | 17 | 5.8 |
|  | Outside the UK | 3 | 1.0 |
| Occupation: | |  |  |
|  | Craft or trades work | 10 | 3.4 |
|  | Manager | 28 | 9.5 |
|  | Plant or machine operator/labour | 3 | 1.0 |
|  | Professional | 66 | 22 |
|  | Service work/care work/sales | 24 | 8.2 |
|  | Technician/clerical | 54 | 18 |
|  | Unemployed/studying | 60 | 20 |
|  | Other work | 49 | 17 |
| Annual income: | |  |  |
|  | Less than £10,000 | 68 | 23 |
|  | £10,000 - £20,000 | 83 | 28 |
|  | £20,000 - 30,000 | 79 | 27 |
|  | £30,000 - £40,000 | 27 | 9.2 |
|  | £40,000 + | 37 | 13 |
| Highest level of education: | | |  |
|  | Did not complete secondary education | 2 | 0.68 |
|  | Completed secondary education | 69 | 23 |
|  | Vocational/trade training | 40 | 14 |
|  | Finished college/6th form | 61 | 21 |
|  | University graduate | 92 | 31 |
|  | Postgraduate qualification(s) | 30 | 10 |
| Musical taste: | |  |  |
|  | Country/Folk | 24 | 8.2 |
|  | Classical | 27 | 9.2 |
|  | Electronic/dance | 13 | 4.4 |
|  | Jazz/blues | 12 | 4.1 |
|  | Pop/rock | 160 | 54 |
|  | R&B/Hip-hop | 19 | 6.5 |
|  | Other | 39 | 13 |
| Political tendencies: | |  |  |
|  | Left (e.g. Green Party) | 28 | 9.5 |
|  | Mid-left (e.g. Traditional Labour Party) | 50 | 17 |
|  | Centre | 56 | 19 |
|  | Mid-right (e.g. Conservative Party) | 54 | 18 |
|  | Right (e.g. UKIP) | 19 | 6.5 |
|  | Not interested | 87 | 30 |
| Choose a preferred hobby/interest from the following list: | | | |
|  | Arts/crafts | 38 | 13 |
|  | Collectibles | 12 | 4.1 |
|  | Music | 53 | 18 |
|  | Reading/literature | 72 | 24 |
|  | Sports | 41 | 14 |
|  | Travel | 50 | 17 |
|  | TV/Radio | 28 | 9.5 |
| Which of the following jokes do you find funniest? | | | |
|  | A guy asks a lawyer what his fee is. “I charge £500 for three questions,” the lawyer says. “That’s awfully expensive, isn’t it?” the guy asks. “Yes,” the lawyer replies, “Now what’s your final question?” | 60 | 20 |
|  | A guy shows up late for work. The boss yells “You should have been here at 8:30!” he replies: “Why? What happened at 8:30? | 75 | 26 |
|  | A lady at a party goes up to Winston Churchill and tells him, “Sir, you are drunk.” Churchill replies, “Madam, you are ugly. In the morning, I shall be sober.” | 114 | 39 |
|  | At the airport they asked me if anybody I didn’t know gave me anything. Even the people I know don’t give me anything. | 24 | 8.2 |
|  | China has a population of a billion people. One billion. That means even if you’re a one in a million kind of guy, there are still a thousand others exactly like you. | 21 | 7.1 |
| Which of the following statements do you agree with most? | | | |
|  | Assisting in the death of a terminally ill friend who is in terrible pain, and who wants to die, is morally permissible. | 131 | 45 |
|  | Before the 3rd month of pregnancy, abortion for any reason (of the mother’s) is morally permissible. | 36 | 12 |
|  | Providing false testimony in court about the whereabouts of a friend who is being charged with murder (i.e., to protect that friend by offering an alibi) is morally permissible. | 7 | 2.4 |
|  | Scientific research on embryonic human stem cells that are the product of in vitro fertilization is morally permissible. | 47 | 16 |
|  | Disagree strongly with all the above statements. | 73 | 25 |
